# Telemedical monitoring of patients with chronic heart failure has a positive effect on total health costs

**DOI:** 10.1186/s12913-018-3070-5

**Published:** 2018-04-10

**Authors:** Robert Herold, Wolfgang Hoffmann, Neeltje van den Berg

**Affiliations:** 1 University Medicine Greifswald, Institute for Community Medicine, Ellernholzstr. 1-2, 17487 Greifswald, Germany; 2 University Medicine Greifswald, Institute for Community Medicine, German Center for Cardiovascular Disease (DZHK), Ellernholzstr. 1-2, 17487 Greifswald, Germany

**Keywords:** Chronic heart failure, Telemedical monitoring, Reimbursement data, Propensity score matching, Health costs

## Abstract

**Background:**

Telemedical programs for patients with chronic heart failure have shown inconsistent effects on survival and hospitalization. Few studies analyzed effects of telemedical interventions on health costs, although this outcome may determine whether or not a successful program will be adopted by health insurance providers. We evaluated a large sized telemedicine program provided by a German statutory health insurance, consisting of regular telephone contacts and, for a subgroup of the participants, provision of an electronic scale in a routine care setting. We examined the effects of the program on the total healthcare costs after one year compared to a matched control group.

**Methods:**

The evaluation was based on reimbursement data of the statutory health insurance. Participants of the program were matched to appropriate controls using a combination of exact (e.g. 5-year age group, gender, NYHA class) and propensity score (e.g. medication, psychiatric comorbidity) matching.

The total health costs after one year were calculated on the basis of regression analyses in an intention-to-treat-approach. In a sensitivity analysis, the subgroup of patients with a documented beginning of the intervention was examined.

**Results:**

Two thousand six hundred twenty two patients with chronic heart failure (55% male, mean age: 73.7 years) were included in the intervention program. 1943 participants (74%) could be matched with appropriate control patients. The telemedicine monitoring program for patients with chronic heart failure reduced total health costs after 12 months of the intervention: − 276€ per quarter year in rural regions and − 18€ in urban regions compared to the control group.

**Conclusions:**

The telemedicine program could reduce total health costs, especially in rural regions in Germany.

## Background

Chronic heart failure (CHF) is a frequent cause of disability, emergency hospital admission and premature death [1, 2]. The prevalence and incidence rates of heart failure increase with age [3]. Due to demographic changes and the increasing probability to survive an acute myocardial infarction and diseases of the heart valves and the myocardium, the number of CHF-patients will continue to increase in the next years [4].

At the same time, chronic heart failure continues to be a fatal disease, with only 35% patients surviving 5 years after the first diagnosis [3]. In 2002, German health insurance companies paid 2.3 times more for patients with than without CHF [5]. Total health costs of CHF in Germany were estimated at 2.9 billion€ in 2006 [6].

Telemedicine programs have been developed to improve monitoring and therapy of patients with chronic diseases [7]. Telemonitoring of patients with chronic heart failure can be used to detect early warning signs of impending decompensation and may be an effective strategy for disease management in high-risk heart failure patients to prevent hospitalizations and mortality [8].

Only a few studies examined the effect of telemedicine programs for CHF patients on total health costs. Systematic reviews report predominantly lower health costs for patients randomized to the intervention group [7–9]. A recent Cochrane review included 41 studies on structured telephone support or telemonitoring programs for patients with CHF. Four of fifteen studies that reported health cost analyses reported reductions in health costs, two studies reported increases in costs [7].

We evaluated the telemonitoring program “AOK-Curaplan Herz Plus” for patients with CHF, offered by a large statutory health insurance in Germany. The program comprised regular telephone coaching by special trained nurses in a telemedicine center, a modem-connected electronic scale in a subgroup of the patients, and the provision of information leaflets about coping with heart failure [10]. The primary outcome of this evaluation was total health costs per quarter year during the first year after enrolment into the intervention program for the entire intervention group (intention to treat analysis) compared to a matched control group. Secondary outcome was total health costs two years after enrolment. Further, a sensitivity analysis was conducted on total health costs per quarter year during the first year after enrolment in the subgroup of patients with a documented begin of the intervention (treated analysis).

## Methods

### Intervention program

AOK-Curaplan Herz Plus is a telemedicine program for patients suffering from CHF, offered by the statutory health insurance AOK Nordost in Germany. The program consists of regular telephone coaching and counseling, provision of information leaflets about disease-related themes, and a telemedical scale for weight monitoring. Telephone coaching and counseling is conducted by trained nurses in a telemedicine service center, specialized on medical services. Telephone contacts include feedback to conspicuous weight increase, talks to patient-individual topics and standard themes (e.g. diet, exercise, adherence to medication) [9]. Telephone contacts are conducted every 4 to 12 weeks, dependent on the patients’ individual needs. If needed, patients can contact the telemedicine service center any time. In case of a deteriorating health situation, the nurses give concrete recommendations or contact the treating physician. The frequency of the telephone calls is individually tailored to each patient. The program is implemented in routine healthcare, additional to usual care, and has no defined duration. AOK-Curaplan Herz Plus started in 2006 and is still running in the German federal states of Berlin and Brandenburg.

Usual care for patients with CHF is based on German and international treatment guidelines.

### Participants

The intervention was implemented in a regular care setting. Patients with a diagnosis of CHF (ICD-10 diagnosis codes I50.12, I50.13 or I50.14 and NYHA class > I) with a high risk for a heart failure related hospitalization were informed about the program and invited to participate. High risk was defined as a prior heart-failure related hospitalization. Exclusion criterion was the presence of any diagnosed mental disorder at the time of recruitment. The recruitment of patients occurred during two phases: from 2006 to 2009, eligible patients were included by their treating general practitioners and cardiologists in private practices. Since 2009, eligible participants were retrieved from the database of the statutory health insurance AOK Nordost and were contacted directly by the health insurance company. All participating patients provided written informed consent.

### Study design

To analyze the effect of the intervention on total health costs compared to an appropriate control group (intention-to-treat analysis), a quasi-experimental design was implemented [11].

Data from 2606 intervention patients and from 205,738 other patients fulfilling the inclusion criteria for the telemedicine program from the time period between the last quarter year of 2006 until the second quarter year of 2012 were retrieved from the database of the statutory health insurance. The data was on a patient-individual level, but completely anonymized, and, except for information about the federal state, without any regional data, so that it was not possible to re-identify single patients. The database included data to all matching variables and outcomes.

**Fig. 1:**
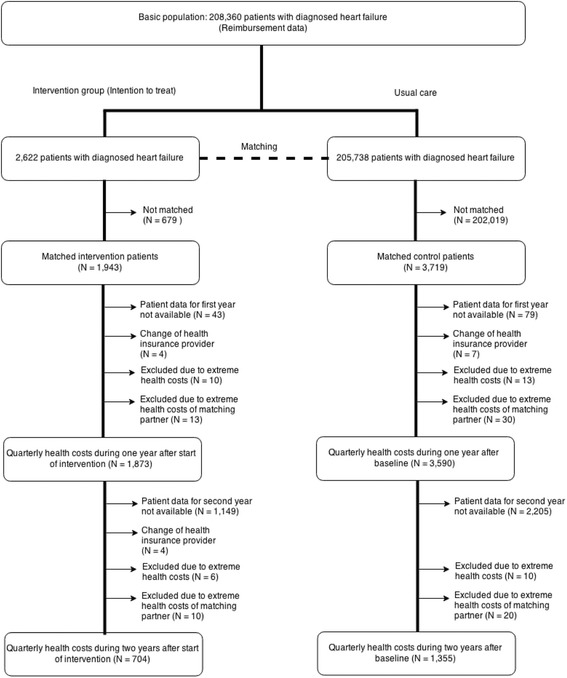
Consort diagram showing the selection of patients to be included in the analysis (intention-to-treat analysis)

The control group was compiled using a combination of exact matching and propensity score matching (see section “matching” for further details). The control patients were retrieved from the total database of the statutory health insurance. For patients of the intervention group, the quarter year of enrollment served as baseline. Figure 1 shows the study design with baseline and follow-up analyses.

In a subsequent sensitivity analysis, the subgroup of patients with a documented start of the intervention was examined (treated analysis). Because of the highly variable time periods between recruitment and start of the intervention throughout the patient group, we conducted a separate matching procedure for the treated analysis using the quarter year of the start of the intervention as baseline quarter year.

Both matching procedures and statistical analyses were based on reimbursement data provided by the AOK Nordost. To analyze the effects of the intervention multivariate regression models were fitted including all matching variables and an array of potential confounders [12].

### Matching procedure

The control group was retrieved from the total database of the statutory health insurance. The base population consisted of all patients with a diagnosis of chronic heart failure. We combined exact and propensity score matching using a nearest neighbor method (greedy algorithm) with restrictions in selected covariates (matching without replace, two controls per case at the maximum) [13].

Intervention patients were matched for their quarter year of enrolment (respectively, the quarter year of starting the intervention in the sensitivity analysis) and controls were drawn dynamically. Dynamically means that controls could be matched to an intervention patient every quarter year as long as they were insured, still alive and not matched in a prior quarter year to another intervention patient.

The following variables had to match exactly: sex, 5-year-age group, NYHA class, number of hospital admissions in the 12 months prior to enrolment, cost category (23 categories) and the presence of any mental or behavioral disorders (ICD-10: F00 – F99). Patients with missing information in any of these variables were excluded from the matching procedure. The following parameters were included as propensity score matching criteria: cardiovascular medication in 6 groups of active agents in the baseline quarter year (angiotensin-converting enzymes, beta-blockers, renin-inhibitors, cardiac glycosides, diuretics, and AT1 receptor blockers) and mental and behavioral disorders in 11 diagnoses groups in the baseline quarter year). Mental illness was considered as a disabling factor for successful program participation. However, a considerable number of patients with mental illness were enrolled. Accordingly, mental disorders had to be included in the matching procedure.

### Exclusion of extreme-cost-patients and capping of high costs

The statutory health insurance uses an algorithm to exclude patients with extreme costs and to cap costs exceeding a defined threshold in their internal analyses to prevent that single outliers bias the results. The underlying assumption is that very high costs are caused by events that are not associated with the disease (e.g. accidents). We followed this algorithm and excluded patients with health costs over 100,000€ per year. Matching partners of high cost patients were also excluded. Additionally, in all remaining patients, inpatient health costs were capped to a maximum of 20,000€ per year, costs for home health care to 15,000€ per year and medication costs to 7000€ per year.

### Statistical analysis

The primary analysis was an intention-to-treat analysis which included all matched patients. After matching, all matched patients received a statistical weight to account for the fact, that for some patients only one matching partner could be assigned (1 for 1943 matched intervention patients, 0.957 for 3552 patients of the control group who were matched to two partners in the intervention group and 1.914 for 167 control patients for whom only one matching partner could be identified).

In the baseline analysis, group differences in continuous variables were compared using the t-test, the chi-square test was used to compare categorical variables.

Primary endpoint of the analysis was the average of the total health costs over the four quarter years of the first year after recruitment. If patients survived less than four quarter years after baseline, we calculated the average of the quarter years when patients were still alive. The analysis of the two-year follow-up proceeded the same way with a maximum of eight quarter years.

The analyses were conducted using multivariate linear regression models. All models were adjusted by all baseline matching variables and by the residential area of the patients (dichotomized as Berlin (= urban) and other (= rural)). An interaction term “study group x residential area” was included in the models because bivariate analyses indicated an effect modification of the intervention of the place of residence on the total health costs.

We considered a *p*-value < 0.05 statistically significant in all comparisons.

Statistics were calculated using R statistical software, version 3.0.0 (R Foundation for Statistical Computing, Vienna, Austria). We used the R packages “MatchIt” [14] for data matching, “survey” [15] for weighted descriptive analyses, and “stats” [16] for regression models.

## Results

### Intention-to-treat analysis

Patients were excluded from the regression analyses for the following reasons: 1) missing follow up data (health cost data were available until the fourth quarter year of 2011), 2) patients changed their health insurance company during follow-up time, 3) extreme health costs above 100,000€/year either of a patient (see methods for details) (Fig. 1).

### Matching

One thousand nine hundred forty three intervention patients (74% of the patients in the intervention group) could be matched to 3719 control patients. Most frequent cause of failing matching was missing information about the NYHA-class (60% of the 679 not matched intervention patients).

### Baseline characteristics

Table 1 shows the distribution of parameter-values for the quarter year of enrolment in the intervention program for patients of the intervention group, and the quarter year of matching for patients of the control group (baseline quarter year). 54% of the patients in both groups were males; the mean age was 74.4 years. NYHA II and III were the most frequent NYHA classes. The total health costs per quarter year in the year prior to the baseline quarter year averaged 3747€ (SD: 3007€) in the intervention group and 3750€ (SD: 3021€) in the control group. 743 patients of the intervention group (38%) had at least one diagnosed mental disorder (ICD-10 Chapter V) in the baseline quarter year.

**Table 1:** Baseline characteristics of the intervention patients and controls

Variable		Mean (SD) / N (%)		Test statistics
		Intervention group (*N* = 1943)	Control group (*N* = 3719)	
Sex	Male	1052 (54.14%)	2014 (54.15%)	–
	Female	891 (45.86%)	1705 (45.85%)	
Age groups	41–45 years	8 (0.41%)	15 (0.41%)	–
	46–50 years	22 (1.13%)	42 (1.12%)	
	51–55 years	46 (2.37%)	88 (2.36%)	
	56–60 years	69 (3.55%)	132 (3.54%)	
	61–65 years	127 (6.54%)	243 (6.52%)	
	66–70 years	305 (15.70%)	585 (15.72%)	
	71–75 years	426 (21.92%)	816 (21.93%)	
	76–80 years	441 (22.70%)	845 (22.73%)	
	81–85 years	314 (16.16%)	601 (16.16%)	
	86–90 years	159 (8.18%)	304 (8.18%)	
	91–95 years	26 (1.34%)	49 (1.33%)	
Mean age [years]		74.44 (SD 8.97)	74.48 (SD 9.04)	*t* = − 0.125, *p* = 0.9004
NYHA class	NYHA I	19 (0.98%)	36 (0.98%)	–
	NYHA II	614 (31.60%)	1175 (31.60%)	
	NYHA III	779 (40.09%)	1491 (40.09%)	
	NYHA IV	531 (27.33%)	1016 (27.33%)	
Hospital admissions	All causes	2.54 (SD 1.73)	2.53 (SD 1.88)	*t* = 0.090, *p* = 0.9283
(baseline year)	Related to CHF	1.37 (SD 0,86)	1.37 (SD 0.86)	–
Emergency hospitalization (baseline year)	All causes	0.65 (SD 1.27)	0.55 (SD 1.30)	*t* = 2.752, *p* = 0.0059**
Total health costs [€] (quarterly mean in baseline year, SD)	All regions	3747 (3007)	3750 (3021)	*t* = −0.037, *p* = 0.9705
	Urban	3723 (2900)	3959 (3503)	*t* = −1.555, *p* = 0.1201
	Rural	3820 (3313)	3677 (2829)	*t* = 0.882, *p* = 0.3777
Health cost [€] (mean, SD)	In-patient	2464 (2376)	2403 (2322)	*t* = 0.896, *p* = 0.3702
	Outpatient	296 (635)	294 (764)	*t* = 0.126, *p* = 0.9000
	Drugs	547 (788)	564 (751)	*t* = − 0.752, *p* = 0.4523
	Remedy	49 (131)	39 (126)	*t* = 2.705, *p* = 0.0068**
	Adjuvant	97 (213)	110 (226)	*t* = −2.100, *p* = 0.0358*
	Home health care	127 (525)	140.06 (426)	*t* = −0.919, *p* = 0.3583
	Travel costs	160 (271)	198 (304)	*t* = −4.660, *p* < 0.0001***
	Rehabilitation	7 (44)	3 (27)	*t* = 3.738, *p* = 0.0002***
Medication (percentage “yes” per active agent in baseline quarter)	Angiotensin-converting enzymes	845 (43.49%)	1637 (44.01%)	Chi^2^ = 0.119, *p* = 0.7301
	Beta-blockers	1261 (64.90%)	2340 (62.93%)	Chi^2^ = 2.057, *p* = 0.1515
	Renin-inhibitors	36 (1.85%)	51 (1.36%)	Chi^2^ = 1.754, *p* = 0.1853
	Cardiac glycosides	279 (14.36%)	548 (14.73%)	Chi^2^ = 0.111, *p* = 0.7388
	Diuretics	1298 (66.80%)	2507 (67.40%)	Chi^2^ = 0.178, *p* = 0.6727
	AT1 receptor blocker	385 (19.81%)	651 (17.51%)	Chi^2^ = 4.397, *p* = 0.0360*
Mental and behavioral disorders	Yes	743 (38.24%)	1421 (38.20%)	–
disorders (baseline quarter)	No	1200 (61.76%)	2298 (61.80%)	
Residential Area	Berlin	1461 (75.19%)	969 (26.06%)	Chi^2^ = 1411.6, *p* < 0.0001***
	Brandenburg	436 (22.44%)	1482 (39.84%)	
	Mecklenburg-Western Pomerania	27 (1.39%)	1235 (33.20%)	
	Other	19 (0.98%)	34 (0.90%)	
Nursing care level of care^a^	No care	1398 (71.95%)	2678 (72.02%)	Chi^2^ = 20.932, *p* = 0.0001***
	Level 1	376 (19.35%)	615 (16.53%)	
	Level 2	159 (8.18%)	370 (9.94%)	
	Level 3	10 (0.51%)	56 (1.52%)	
Living in nursing home	Yes	159 (8.18%)	498 (13.39%)	Chi^2^ = 33.182, *p* < 0.0001***
	No	1784 (91.82%)	3221 (86.61%)	

^a^Standardized classification of the level of nursing care in Germany, Significance codes: ***p < 0.001, **p < 0.01, *p < 0.05

Intervention and control group were similar in most variables. Significant differences concerned the medication with AT1 receptor blockers, the number of emergency hospitalizations, the residential area, the level of care, and the proportion of patients living in a nursing home.

### One-year follow-up

Figure 1 shows the flowchart of the analysis; 5433 of 5662 patients could be included in the one-year-follow-up analysis. The processed mean average quarterly health costs in the first year following the recruitment the total health care costs differ by 8€ overall between the intervention (2921€) and control (2929€) groups. Comparing the health costs of various subgroups of patients reveals considerable differences. As expected, costs are much higher in the group of patients who died in the year after recruitment. Rural patients caused lower health costs in both groups compared to the patients in Berlin. The crude difference between intervention and control patients was − 64€ in Berlin and − 259€ in rural regions, indicating effect modification which needed to be appropriately addressed in the fully adjusted model. Average health costs in the first year after baseline were broken down to cost segments. Inpatient costs contribute the largest part of the total health costs in both groups (Table 2).

**Table 2:** Unadjusted subgroup means of processed^a^ quarterly health costs in the first year after baseline^b^

	Intervention group		Control group	
	Mean (CI 95%)	N	Mean (CI 95%)	N
All Patients	€ 2921 (2773–3068)	1873	€ 2929 (2816–3041)	3590
Survived first year	€ 2397 (2279–2515)	1670	€ 2417 (2320–2514)	3105
Died	€ 7229 (6509–7948)	203	€ 6176 (5728–6625)	485
Male	€ 2941 (2740–3142)	1004	€ 3013 (2854–3172)	1925
Female	€ 2897 (2679–3115)	869	€ 2830 (2673–2987)	1665
Urban (Berlin)	€ 3023 (2845–3200)	1405	€ 3086 (2844–3329)	930
Rural	€ 2615 (2360–2869)	468	€ 2873 (2748–2999)	2660
Total health costs	€ 2921 (2773–3068)	1873	€ 2929 (2816–3041)	3590
Inpatient	€ 1557 (1441–1673)	1873	€ 1500 (1419–1580)	3590
Outpatient	€ 364 (322–405)	1873	€ 346 (309–382)	3590
Drugs	€ 508 (487–529)	1873	€ 531 (515–547)	3590
Remedy	€ 57 (50–63)	1873	€ 47 (42–51)	3590
Adjuvant	€ 115 (104–126)	1873	€ 133 (124–142)	3590
Home health care	€ 155 (134–176)	1873	€ 179 (163–194)	3590
Travel costs	€ 160 (142–178)	1873	€ 193 (176–209)	3590
Rehabilitation	€ 5 (3–6)	1873	€ 2 (1–3)	3590

^a^Health costs after performing the excluding-and-capping algorithm

^b^Maximally 4 quarter years, less for patients who died within first year

To represent the modification of the intervention effect by place of residence of the patients (urban or rural) we added the interaction term “study group x residential area” into the regression model. Table 3 shows the regression model for total health costs in the first year of follow up. This model was adjusted for all matching variables, the patients’ residential area and the interaction between study group and residential area. Statistically significant determinants for lower health costs were the intake of a beta-blocker (*p* = 0.0014). Significant predictors for higher health costs were higher baseline health costs (*p* < 0.0001), the intake of diuretics (*p* < 0.0001) a diagnosis in the group of organic, mental disorders (F00 – F09, *p* = 0.0018).

**Table 3:** Results of regression analysis of processed^a^ quarterly health costs in first year after baseline (*N* = 5463)

	Reference	Estimate [in €]	CI 95%		*P*-value
			2.5%	97.5%	
Study group	Control group	−276.04	− 573.18	21.10	0.0686
Residential area (Berlin vs. rural)	Rural	180.81	−49.03	410.65	0.1231
Interaction: Group x Residential area		257.77	−131.35	646.90	0.1941
Age group					
46–50 years	41–45 years	878.30	− 652.97	2409.58	0.2609
51–55 years	41–45 years	669.75	− 749.92	2089.43	0.3551
56–60 years	41–45 years	641.50	−743.15	2026.14	0.3638
61–65 years	41–45 years	448.94	− 905.54	1803.43	0.5159
66–70 years	41–45 years	828.90	−502.34	2160.14	0.2223
71–75 years	41–45 years	1117.78	− 210.13	2445.69	0.0990
76–80 years	41–45 years	1160.55	− 167.31	2488.41	0.0867
81–85 years	41–45 years	973.17	− 359.70	2306.04	0.1524
86–90 years	41–45 years	876.95	− 471.87	2225.77	0.2025
91–95 years	41–45 years	1168.45	− 340.20	2677.11	0.1290
Sex	Male	−73.85	−245.19	97.49	0.3982
NYHA class					
*NYHA II*	NYHA I	18.38	− 793.15	829.91	0.9646
*NYHA III*	NYHA I	210.53	−600.60	1021.65	0.6109
*NYHA IV*	NYHA I	398.18	−419.09	1215.45	0.3396
Hospitalizations related to CHF	(continuous)	52.03	−51.72	155.79	0.3256
Baseline health costs	(continuous)	0.37	0.34	0.40	< 0.0001***
Intake of medication related to CHF (yes / no by groups of agents)					
Angiotensin-converting enzyme	no	− 139.92	−313.52	33.69	0.1142
Beta-blocker	no	−279.06	− 450.13	− 107.98	0.0014**
Renin-inhibitors	no	−75.07	− 734.15	584.01	0.8233
Cardiac glycosides	no	147.81	−81.40	377.01	0.2062
Diuretics	no	394.65	214.89	574.40	< 0.0001***
AT1 receptor blocker	no	−25.55	− 247.64	196.54	0.8216
Mental and behavioral disorders (yes / no by blocks of ICD-10 Chapter V)					
F00 – F09 (Organic, including symptomatic, mental disorders)	no	465.72	173.79	757.65	0.0018**
F10 – F19 (Mental and behavioral disorders due to psychoactive substance use)	no	39.64	− 257.67	336.94	0.7938
F20 – F29 (Schizophrenia, schizotypal and delusional disorders)	no	−34.52	− 936.45	867.41	0.9402
F30 – F39 (Mood [affective] disorders)	no	109.85	−120.77	340.48	0.3504
F40 – F49 (Neurotic, stress-related and somatoform disorders)	no	51.25	− 188.92	291.42	0.6757
F50 – F59 (Behavioral syndromes associated with physiological disturbances and physical factors)	no	343.92	− 130.81	818.64	0.1556
F60 – F69 (Disorders of adult personality and behavior)	no	−120.35	− 859.94	619.25	0.7497
F70 – F79 (Mental retardation)	no	321.91	−921.90	1565.71	0.6119
F80 – F89 (Disorders of psychological development)	no	–	–	–	–
F90 – F98 (Behavioral and emotional disorders with onset usually occurring in childhood and adolescence)	no	612.00	− 991.08	2215.09	0.4542
F99 – F99 (Unspecified mental disorders)	no	490.90	− 1333.73	2315.52	0.5979
Intercept	–	249.86	− 1318.15	1817.88	0.7548

^a^Health costs after performing the excluding-and-capping algorithm, Significance codes: ***p < 0.001, **p < 0.01, *p < 0.05

Compared to the matched controls, total health costs in the first year after baseline were lower in the intervention group. The difference between both groups was − 276€ in rural regions and − 18€. Neither the parameters for the study group nor for the residential area and the interaction between them were statistically significant.

### Two-year follow-up

Table 4 shows the regression model for the two-year follow up (sensitivity analysis planned a priori). The model was adjusted for the same parameters as the model for the one-year-follow up. Statistically significant determinant for lower health costs was the intake of beta blockers (*p* = 0.0007). Higher baseline health costs (*p* < 0.0001) and the intake of diuretics (*p* = 0.0003) were significant determinants for higher health costs. The intervention group showed lower health costs in rural regions (− 299€ per quarter year) but higher health costs in Berlin (+ 109€, compound effect of study group (− 299€) and interaction of group and residential area (+ 407€)) compared to the control group.

**Table 4:** Results of regression analysis of processed^a^ quarterly health costs in first two years after baseline (*N* = 2059)

	Reference	Estimate	CI 95%		*p*-value
			2.5%	97.5%	
Study group	Control group	−298.58	− 1312.13	714.97	0.5635
Residential area (Berlin vs rural)	Rural	262.69	− 101.60	626.98	0.1575
Interaction: Group x Residential area		407.35	407.35	1493.06	0.4619
Age group					
46–50 years	41–45 years	2832.23	− 782.80	6447.27	0.1246
51–55 years	41–45 years	2875.11	−606.67	6356.89	0.1055
56–60 years	41–45 years	2600.31	−823.07	6023.69	0.1365
61–65 years	41–45 years	2569.53	−820.65	5959.71	0.1373
66–70 years	41–45 years	2840.77	− 533.57	6215.11	0.0989
71–75 years	41–45 years	3259.24	− 109.66	6628.14	0.0579
76–80 years	41–45 years	3318.69	− 50.93	6688.32	0.0536
81–85 years	41–45 years	3017.17	−356.79	6391.14	0.0796
86–90 years	41–45 years	2919.52	− 469.23	6308.27	0.0913
91–95 years	41–45 years	2870.68	− 689.65	6431.00	0.1140
Sex	Male	−96.52	− 369.46	176.42	0.4881
NYHA class					
*NYHA II*	NYHA I	314.97	−757.31	1387.24	0.5646
*NYHA III*	NYHA I	375.11	− 696.74	1446.96	0.4926
*NYHA IV*	NYHA I	776.31	−302.57	1855.18	0.1584
Hospitalizations related to CHF	(continuous)	−27.61	− 195.63	140.40	0.7472
Baseline health costs	(continuous)	0.34	0.29	0.39	< 0.0001***
Intake of medication related to CHF (yes / no by groups of agents)					
Angiotensin-converting enzyme	no	−251.08	− 520.92	18.76	0.0682
Beta-blocker	no	− 470.81	− 741.91	−199.71	0.0007***
Renin-inhibitors	no	−283.79	− 1553.61	986.03	0.6612
Cardiac glycosides	no	97.57	− 252.61	447.75	0.5848
Diuretics	no	533.86	247.94	819.78	0.0003***
AT1 receptor blocker	no	−140.25	−491.33	210.83	0.4335
Mental and behavioral disorders (yes / no by blocks of ICD-10 Chapter V)					
F00 – F09 (Organic, including symptomatic, mental disorders)	no	386.31	−87.09	859.70	0.1097
F10 – F19 (Mental and behavioral disorders due to psychoactive substance use)	no	−83.55	− 544.49	377.39	0.7223
F20 – F29 (Schizophrenia, schizotypal and delusional disorders)	no	− 288.83	− 1375.19	797.53	0.6021
F30 – F39 (Mood [affective] disorders)	no	30.78	− 326.67	388.23	0.8659
F40 – F49 (Neurotic, stress-related and somatoform disorders)	no	11.66	− 351.60	374.92	0.9498
F50 – F59 (Behavioral syndromes associated with physiological disturbances and physical factors)	no	−86.95	− 775.45	601.54	0.8044
F60 – F69 (Disorders of adult personality and behavior)	no	816.43	− 367.37	2000.23	0.1764
F70 – F79 (Mental retardation)	no	291.70	− 2155.13	2738.54	0.8152
F80 – F89 (Disorders of psychological development)	no	–	–	–	–
F90 – F98 (Behavioral and emotional disorders with onset usually occurring in childhood and adolescence)	no	−340.42	− 2933.64	2252.80	0.7969
F99 – F99 (Unspecified mental disorders)	no	5878.45	− 562.31	12,319.21	0.0736
Intercept	–	− 1689.37	− 5226.05	1847.32	0.3490

^a^Health costs after performing the excluding-and-capping algorithm, Significance codes: ***p < 0.001, **p < 0.01, *p < 0.05

### Sensitivity analysis (treated patients)

In a sensitivity analysis, the total health costs per quarter year of patients with a documented start of treatment were compared to a separately matched control group. Here, only the one-year follow-up was analyzed because data of a large part of the participants were not available for two-year follow-up. The intervention group showed lower health costs in rural regions (− 551€ per quarter year) and in urban regions (− 267€, compound effect of study group (− 551€) and interaction of group and residential area (+ 284€)) as well.

## Discussion

In an analysis based on routine data, collected for reimbursement purposes, it was shown that the total health costs for intervention patients after one year were lower compared to a control group that had been matched for a large array of variables. Beside age, NYHA-class and baseline health costs, somewhat unexpectedly, the place of residence of the participating patients (urban or rural) was an important determinant for the development of the health costs over time (generally with better development for patients with a rural place of residence). A possible cause could be the higher availability of health care services in urban areas that tend to be more cost-intensive at the same time.

In addition to the intention-to-treat analysis, we performed a treated analysis with patients for which the beginning of the intervention was positively documented. The results of the treated analysis support the findings of the more conservative intention-to-treat approach.

Hendricks et al. analyzed inpatient treatment costs of 1202 patients participating in a telemedicine monitoring program from a large German statutory health insurance company. The follow-up time was 54 months. The average annual costs for CHF related inpatient treatment in the intervention group were significantly lower than the equivalent costs in the control group (684€ per patient, Mann-Whitney-U-Test: *p* < 0.0001). Both groups show no significant differences in respect of number of hospital days and average costs per hospital stay [17].

In a prospective study on inpatient costs, 502 patients were randomized into an intervention and a control group. The intervention consisted of the monitoring of the body weight of the participants during one year. After an average observation period of 12 months, the annual hospital costs were reduced by 7128€ per patient while annual drug expenditure had increased by 245€ per patient (not significant). The total treatment costs had decreased by 6993€ per patient (*p* = 0.05). Reported savings are much greater compared to our results. But unfortunately, the authors don’t adjust the results for baseline differences [18].

Neumann et al. evaluated the results of the HeartNetCare-HF (HNC) study (Würzburg / Germany). The overall costs per person were 3535€ in the HNC intervention group (*n* = 352) and 3038€ in the usual group (*n* = 363) within six months (*p* value for difference: 0.10). The HNC group showed a reduced risk of all-cause death (9% vs. 14%, *p* = 0.03). The authors state a positive cost-effectiveness (the ratio between the difference in costs and the difference in all-cause mortality) since HNC accounted for 8284€ per death avoided within the 6 months [19].

Zertiva is a telemedicine program for patients with CHF, offered by the statutory health insurance “Techniker Krankenkasse”. 164 Patients were included after they had been hospitalized due to CHF; the follow-up period was 180 days. The standard care cohort was retrieved from routine data. The telemedicine group showed lower total health costs after 180 days compared to the matched controls (2292€ per patient and 3746€, respectively). Effectiveness adjusted health costs (total health costs divided by “success rate”, meaning no hospitalization due to CHF during follow-up period) accounted for 3065€ for the telemedicine group and 6397€ for the control group, respectively [20].

In a retrospective analysis, conducted on the basis of data from 2009 until 2013 of a comprehensive telehealth program (existing of the transmission of vital data, daily telephone coaching, and continuous decision-making support) the cost data of 575 patients with chronic cardiovascular diseases were compared to the data of 1178 matched patients. The monthly average medical costs were US$588 (SD 1498) in the telehealth group and US$1164 (SD 3037) in the matched control group (*p* = 0.02) [21]. This study was conducted in Taiwan and results might less comparable to our study.

In many studies on telemonitoring for CHF patients, survival and the number of hospitalizations are endpoints. The effects of telemonitoring on health costs have been analyzed only in few studies. Often, only inpatient health costs instead of total health costs were considered as endpoint. Previous studies differ in design (randomized controlled design vs. use of routine data for comparison), average age, NYHA stage at baseline, and follow-up time. However, in most previous studies intervention groups tend to show reductions in health costs. Compared to our intervention patients, other study groups tend to be younger at baseline, have a lower NYHA class and have a lower number of participants. Moreover, other settings tend to be more specific, included patients seem to less heterogeneous and therefore less reflecting the total group of patients.

### Strengths and limitations

A strength of this study is the large number of participating patients and its closeness to regular health care. All data used for the analysis were retrieved from original reimbursement data. Hence, there was no systematic difference in the data structure or data assessment between the patients in the intervention, and in the control group. The documentation of total direct health costs was complete and valid comparisons could be calculated. Since the intervention was conducted in a routine care setting, the results are likely transferable to the entire non-institutionalized CHF population of this statutory health insurance.

The study has several limitations. By applying propensity score matching we can only approximate the advantages of randomized trials. Data on social demographic variables were not available and despite a comprehensive two step matching process some residual confounding may have persisted due to unobserved variables. We could only match patients whose latest diagnose of NYHA class was within the timeframe of the available data (but before the date of matching).

A large part of the patients did not meet the inclusion and exclusion criteria because they had a diagnosed mental disorder at baseline. Possible causes were 1) participating cardiologists were not aware of diagnoses of mental disorders with their patients, 2) exact dates of diagnoses are not available in the data base, the data is stored on the basis of quarter years, and 3) data is reported retrospectively by the providers of medical services.

The large number of patients with mental disorders may have had an influence on the effect size of the intervention. On the other hand, including patients with mental disorders meets the real composition of the patient group and increases the external validity of the results.

The follow-up time is relatively short. Results might be inflated [22, 23] since long term effects like regression to the mean could not be analyzed. Interventions to maintain good adherence for long-term lifestyle changes can be very challenging [24]. The health insurance could not provide information on the number of telephone contacts per patient. As part of the program some patients had received additional services (such as provision of a modem-connected scale), but this was unknown for the individual patients. Finally, only data of one health insurance were available, therefore the results can only be representative for the member population of this statutory health insurance, not for the entirety of CHF patients.

### Implications of telemonitoring programs for the treatment of CHF patients

The analysis of the telemonitoring program of the AOK Nordost and the results of other programs show that telemonitoring programs do not increase costs of treatment. This opens good opportunities to integrate telemonitoring programs in the treatment and monitoring of patients with CHF. Especially in rural regions with large distances to providers of medical services, this kind of intervention can ensure or improve the quality of healthcare.

## Conclusions

The AOK-Curaplan Herz Plus program reduced short-term health costs, especially in rural regions. The sensitivity analysis (treated approach) shows larger effects which is an indication that the effects do really originate from the telemedicine intervention and that the true impact might be larger than observed in the primary intention-to-treat-analysis.

## Abbreviations

CHF: Chronic heart failure
HNC: HeartNetCare-Heart Failure Study
NYHA: New York Heart Association

## References

[1] Statistisches Bundesamt, editor. Diagnosedaten der Patienten und Patientinnen in Krankenhäusern - Fachserie 12 Reihe 6.2.1. Wiesbaden; 2012. p. 103.

[2] Statistisches Bundesamt, editor. Todesursachen in Deutschland - Fachserie 12 Reihe 4. Wiesbaden; 2013. p. 53.

[3] Mozaffarian D, Benjamin EJ, Go AS, Arnett DK, Blaha MJ, Cushman M (2016). Heart disease and stroke statistics—2016 update. Circulation.

[4] Roger VL (2013). Epidemiology of heart failure. Circ Res.

[5] Zugck C, Müller A, Helms TM, Wildau HJ, Becks T, Hacker J (2010). Gesundheitsökonomische Bedeutung der Herzinsuffizienz: Analyse bundesweiter Daten. Dtsch Med Wochenschr.

[6] Neumann T, Biermann J, Erbel R, Neumann A, Wasem J, Ertl G (2009). Heart failure: the commonest reason for hospital admission in Germany: medical and economic perspectives. Dtsch Arztebl Int.

[7] Inglis SC, Clark RA, Dierckx R, Prieto-Merino D, Cleland JG. Structured telephone support or non-invasive telemonitoring for patients with heart failure. Cochrane Database Syst Rev. 2015;(10):CD007228. 10.1002/14651858.CD007228.pub3 PMC8482064 26517969

[8] Chaudhry SI, Phillips CO, Stewart SS, Riegel B, Mattera JA, Jerant AF (2007). Telemonitoring for patients with chronic heart failure: a systematic review. J Card Fail.

[9] Clark RA, Inglis SC, McAlister FA, JGF C, Stewart S (2007). Telemonitoring or structured telephone support programmes for patients with chronic heart failure: systematic review and meta-analysis. BMJ.

[10] Gesellschaft für Patientenhilfe DGP mbH. Leistungsmerkmale von Cordiva [Internet]. 2009. Available from: http://www.cordiva.de/de/html/cordiva-programmbeschreibung.html. Accessed 4 Apr 2018.

[11] Stuart EA, Rubin DB, Osborne JW (2007). Best practices in quasi-experimental designs: matching methods for causal inference. Best practices in quantitative social science.

[12] Ho DE, Imai K, King G, Stuart EA (2007). Matching as nonparametric preprocessing for reducing model dependence in parametric causal inference. Polit Anal.

[13] Rubin DB (1973). Matching to remove Bias in observational studies. Biometrics.

[14] Ho DE, Imai K, King G, Stuart EA. MatchIt: nonparametric preprocessing for parametric causal inference. J Stat Softw. 2011;42(8)

[15] Lumley T (2004). Analysis of complex survey samples. J Stat Softw.

[16] Core Team R. R: A language and environment for statistical computing. Vienna; 2015.

[17] Hendricks V, Schmidt S, Vogt A, Gysan D, Latz V, Schwang I (2014). Case management program for patients with chronic heart failure: effectiveness in terms of mortality, hospital admissions and costs. Dtsch Arztebl Int.

[18] Kielblock B, Frye C, Kottmair S, Hudler T, Siegmund-Schultze E, Middeke M (2007). Impact of telemetric management on overall treatment costs and mortality rate among patients with chronic heart failure. Dtsch med Wochenschr.

[19] Neumann A, Mostardt S, Biermann J, Gelbrich G, Goehler A, Geisler BP (2014). Cost-effectiveness and cost-utility of a structured collaborative disease management in the interdisciplinary network for heart failure (INH) study. Clin Res Cardiol.

[20] Heinen-Kammerer T, Kiencke P, Motzkat K, Nelles S, Liecker B, Petereit F, Kirch W, Badura B (2006). Telemedizin in der Tertiärprävention: Wirtschaftlichkeitsanalyse des Telemedizin-Projektes Zertiva bei Herzinsuffizienz-Patienten der Techniker Krankenkasse. Prävention.

[21] Ho YL, Yu JY, Lin YH, Chen YH, Huang CC, Hsu TP (2014). Assessment of the cost-effectiveness and clinical outcomes of a fourth-generation synchronous telehealth program for the management of chronic cardiovascular disease. J Med Internet Res.

[22] Ioannidis JPA (2008). Why most discovered true associations are inflated. Epidemiology.

[23] Krum H, Tonkin A (2003). Why do phase III trials of promising heart failure drugs often fail? The contribution of “regression to the truth”. J Card Fail.

[24] Friedman LM, Furberg CD, Demets DL. Participant adherence. Fundamentals of Clinical Trials. 2010:251–68.

